# Shoot–Root Interplay Mediates Defoliation-Induced Plant Legacy Effect

**DOI:** 10.3389/fpls.2021.684503

**Published:** 2021-08-05

**Authors:** Xiliang Li, Zhen Zhang, Fenghui Guo, Junjie Duan, Juan Sun

**Affiliations:** ^1^Institute of Grassland Research, Chinese Academy of Agricultural Sciences, Hohhot, China; ^2^College of Grassland Science, Qingdao Agricultural University, Qingdao, China; ^3^College of Grassland Science, Shanxi Agricultural University, Taigu, China

**Keywords:** phenotypic plasticity, plant functional trait, legacy effect, allometric scaling, shoot-root interaction, defoliation, grassland

## Abstract

Shoot defoliation by grazers or mowing can affect root traits of grassland species, which may subsequently affect its aboveground traits and ecosystem functioning (e.g., aboveground primary production). However, experimental evidence for such reciprocal feedback between shoots and roots is limited. We grew the perennial grass *Leymus chinensis*–common across the eastern Eurasian steppe–as model species in a controlled-hydroponics experiment, and then removed half of its shoots, half of its roots, or a combination of both. We measured a range of plant aboveground and belowground traits (e.g., phenotypic characteristics, photosynthetic traits, root architecture) in response to the shoot and/or root removal treatments. We found the regenerated biomass was less than the lost biomass under both shoot defoliation and root severance, generating a under-compensatory growth. Root biomass was reduced by 60.11% in the defoliation treatment, while root severance indirectly reduced shoot biomass by 40.49%, indicating a feedback loop between shoot and root growth. This defoliation-induced shoot–root feedback was mediated by the disproportionate response and allometry of plant traits. Further, the effect of shoot defoliation and root severance on trait plasticity of *L. chinensis* was sub-additive. That is, the combined effects of the two treatments were less than the sum of their independent effects, resulting in a buffering effect on the existing negative influences on plant persistence by increased photosynthesis. Our results highlight the key role of trait plasticity in driving shoot–root reciprocal feedbacks and growth persistence in grassland plants, especially perennial species. This knowledge adds to earlier findings of legacy effects and can be used to determine the resilience of grasslands.

## Introduction

Grasslands that are dominated by perennial species, which cover the largest terrestrial land area worldwide (Gibson, 2009), are commonly used by livestock grazing or mowing for haymaking and provide essential services for human (Dixon et al., 2014; Fetzel et al., 2017). In recent decades, a pressing challenge for ecologists is to understand the impacts of intensifying land use on biodiversity conservation and ecosystem dynamics, in particular, the overgrazing and frequent mowing of grasslands (Lü et al., 2012; Wang B. et al., 2020). With both grazing and mowing, the removal of plant shoot tissue (i.e., “defoliation”) is the main mechanism underlying the effects on plant performance and ecosystem function of grasslands (Liu et al., 2015; Lezama and Paruelo, 2016). Numerous studies have described the short-term responses to defoliation, these include individual growth (Ferraro and Oesterheld, 2002; Zhang Z. et al., 2020), physiological-biochemical characteristics (Loaiza et al., 2017; Liu et al., 2019), and biomolecular processes (Wan et al., 2015). However, knowledge on the legacy effects of defoliation (i.e., indirect responses) is limited, and is essential to improving plant persistence and grassland sustainability (Tsuzuki et al., 2020).

Legacy effect is defined as the persistence of impacts of a certain ecological event on ecosystem processes after the activity ceased (Cuddington, 2011; Kaisermann et al., 2017). Since the early 1990s, a growing number of studies have examined legacy effects in the field during ecosystem succession, plant invasion, ecosystem engineering, and human-induced land use change (Cuddington, 2011). Possible mechanisms of legacy effects on plant performance detected in previous studies may broadly involve either one or three major drivers: soil abiotic properties (Barthelemy et al., 2019), feedback of soil microbiome (Veen et al., 2014; Wang X. et al., 2020), and/or plant maternal effects (Ren et al., 2017; Yin et al., 2020). First, soil abiotic factors, such as nutrient availability, pH, and physical properties of soil substrates, can be influenced by plant species or ecological events and strongly affect plant performance (Chen Q. et al., 2018; Barthelemy et al., 2019). Second, the microbiome of plant tissues and soil are sensitive to external disturbance and can produce strong effects on plant performance via plant–soil and plant–phyllosphere feedback (Whitaker et al., 2017; Chen T. et al., 2018). Third, plant maternal effect mediated by epigenetic inheritance and changes in seed or bud quality is essential for subsequent plant growth and plant species resilience (Ren et al., 2017; Rendina González et al., 2018; Yin et al., 2019). In natural grasslands, plant communities are commonly dominated by perennial species (Benson and Hartnett, 2006). Therefore, in addition to the three major drivers described above, the feedback loop between shoot and root plasticity may be essential for determining the growth and persistence of perennial plant species.

Defoliation-related trait plasticity plays a central regulatory role in plant fitness, population development, and ecosystem function (Cruz et al., 2010; Leuschner et al., 2013; Li et al., 2015). The mechanism underlying the trait plasticity is the so-called compensation effect, in which plants can accelerate their growth to compensate the losses in performance induced by defoliation (Anten et al., 2003; van Staalduinen and Anten, 2005). However, in response to defoliation, plants can have under-, equal-, and over-compensatory growth according to the quantitative relationship between regenerated and removed biomass (van der Graaf et al., 2005; Ma et al., 2020). In consequence, the magnitude of phenotypic plasticity is dependent on defoliation intensity, and shifts in these traits may alter photosynthetic function and the regeneration capacity of tissue (Shen et al., 2019; Wang et al., 2019; Yuan et al., 2020). Additionally, defoliation may also influence the root architecture and biomass production mediated by photosynthate reallocation (Liu et al., 2018). It is well known that high-intensity defoliation would be more detrimental for root growth (Dawson et al., 2004). Meanwhile, some studies suggested that the responses of leaf and root traits related to defoliation are uncoupled (Kirkegaard et al., 2015; Erktan et al., 2018). These uncertainties in the root response may be due to the variations in species and the kind of functional traits measured (Husáková et al., 2018). Considering the feedback loop between shoot and root, our understanding on how shoot performance is affected by defoliation-induced root plasticity remains limited.

Plant traits can reflect the general response and trade−offs (or coordination) of the ecological function of environmental fluctuations (Abalos et al., 2018; van der Merwe et al., 2021). Thus, a trait-based approach allows a better understanding of how plants respond to external disturbance (Garnier and Navas, 2012). We conducted a controlled-hydroponics experiment to investigate the interactions between shoot defoliation and root severance of *Leymus chinensis* and their effects on its shoot and root traits. The perennial grass *L. chinensis* was selected as the focal species because it is a common forage species and has widely distributed across the eastern Eurasian temperate steppe (Liu et al., 2018). Specifically, to investigate defoliation-induced legacy effects on plant performance due to the changes in root trait, we asked three questions: (1) How do functional traits of shoots and roots differentially respond to defoliation? (2) How do shoot performances feedback to root plasticity? (3) Is the effect of shoot defoliation and root severing on trait variation additive or non-additive? Answering these questions can improve our understanding of the grazing stress tolerance and interannual stability of this important forage species.

## Materials and Methods

### Focal Species

*Leymus chinensis* is a perennial species in the family Gramineae possessing a rhizomatous root system and largely reproduces via clonal buds, leading to its extensive spread, through which it often forms large monocultures (Bai et al., 2009). It is abundant in many important grazing ecosystems across the eastern Eurasian steppe (Li et al., 2015), which includes the outer Baikal area of Russia, the People’s Republic of Mongolia, and the northern grasslands of China. Owing to its excellent stress tolerance, *L. chinensis* is found across a broad temperature and precipitation gradient. In addition, it is highly palatable to grazing livestock and is frequently used to make hay. *L. chinensis* is also a desirable species for reseeding in degraded grassland because of its rapid rhizomatous propagation (Liu and Han, 2008).

### Experimental Design

The experiment was conducted in a growth chamber, and *L. chinensis* was grown via the hydroponics. This method allows us to remove roots easily and to detect how simulated root plasticity influence the plant performance. The following four treatments were used in our experiment (Supplementary Figure 1): (i) control (CK); (ii) shoot defoliation (SD_1/2cut_, 50% leaf removal); (iii) root severance (RS_1/2cut_, removal of 50% of the roots from the base of the root system); and (iv) shoot defoliation and root severance (SR_1/2cut_ removal of 50% of leaves and roots).

For our experiment, *L. chinensis* seeds were from the same genotype and collected from a well-watered common garden at the Chinese Academy of Agricultural Sciences’ Shaerqin Research Station (40°34′N, 111°56′E, 1,040 m a.s.l.) at Hohhot, Inner Mongolia of China, to ensure that their maternal environment was consistent. Plump seeds were surface-sterilized by soaking in 2% sodium hypochlorite (*w*/*v*) for 25 min. *L. chinensis* seeds germinated approximately 10 days after sowing each seed into 9 cm deep soil plugs. To ensure consistency, we selected healthy, 20-day-old individuals with the same initial growth and development status for the hydroponics experiment with Hoagland nutrient solution (Ren et al., 2017). In each hydroponics container (10-cm diameter × 20-cm height), we transplanted four *L. chinensis* individuals and arranged them symmetrically along the edge of the containers. The hydroponics experiments were conducted in a plant growth chamber set to a photoperiod of 14 h/10 h (light/dark), temperatures of 25°C in the light and 15°C in the dark phases, and a light intensity of 500 μmol m^–2^ s^–1^. The nutrient solution was replaced every 3 days throughout the duration of the experiment.

In total, we conducted two cycles’ of shoot defoliation and root severance treatments. Specifically, the first and second treatment cycles were conducted on the 15th and 30th day after transplanting *L. chinensis* seedlings, respectively, for the SD_1/2cut_, RS_1/2cut_, and SR_1/2cut_ treatments. For the shoot defoliation treatment, half of each leaf was cut off along the middle of the leaf (i.e., removing half of the leaf widthwise) using a pair of scissors (Supplementary Figure 1). For the root severance treatment, the fibrous roots of each *L. chinensis* individual were divided into two symmetrical halves and randomly removed a selected half (Supplementary Figure 1). During the experiment, the removed leaves and roots of *L. chinensis* were collected and oven-dried at 70°C for 72 h for the calculation of biomass accumulation.

### Measurements

During the second treatment cycle, we measured the vertical height, relative chlorophyll value, and photosynthetic traits (e.g., net photosynthetic rate and leaf respiration rate) of leaves every 5 days; that is, in total, these parameters were measured four times. At the end of the second treatment cycle (45 days after transplanting), we measured leaf and root phenotypic traits (e.g., total root length, total root surface area) of *L. chinensis*. Roots and shoots of 65-day-old experimental *L. chinensis* (20 days after germination in soil plugs and 45 days in hydroponic containers) were separately harvested and oven-dried at 70°C for 72 h for subsequent measurement of biomass.

To measure the net photosynthetic rate, a LI–6400XT portable photosynthesis system (LI-COR, Lincoln, NE, United States) was used at light saturation (1,000 μmol m^–2^ s^–1^) (Ren et al., 2017). The respiration rates of leaves were measured at 0 μmol m^–2^ s^–1^ after 30 min of zero irradiance on the same leaf (Ayub et al., 2014). We calculated the gross photosynthetic rate as the difference between the value of the net photosynthetic rate and the respiration rate. The relative chlorophyll values were measured using a SPAD-502 Plus portable chlorophyll meter (Konica Minolta Inc., Japan).

Using the portable leaf area meter CI-202 (CID, Walz, Camas, WA, United States), leaf phenotypic traits, such as leaf area, leaf length, leaf width, and leaf perimeter, were measured. Each measured leaf was precisely weighted to 0.1 mg accuracy (Shimadzu Inc., Japan). Additionally, root morphology, including total root length, total root surface area, total root volume, average root diameter, root tip number, root tip forks, and root tip crossings were measured using a WinRhizo^TM^ scanner-based system (Regent Instruments Inc., Quebec, ON, Canada). The samples showed no signs of fragmentation because the use of hydroponic containers enabled the harvest of intact root systems.

### Calculation and Statistics

In the CK, SD_1/2cut_, RS_1/2cut_, and SR_1/2cut_ treatments, the growth rates were represented by the average slopes of plant height along a time series using a linear regression analysis. The accumulated biomass of shoots and roots were the sums of the remaining biomass and the corresponding mass of removed leaves and roots. We calculated specific leaf area using the recorded single leaf mass and leaf area. Similarly, the specific root length was calculated using root biomass and total root length. In addition, respiration consumption was the proportion of the respiration rate in the gross photosynthetic rate of leaves.

The extent of trait variation within each treatment was represented by the coefficient of variation of trait values in each of the shoot and root traits (Zhang B. et al., 2020). In addition, to compare the sensitivity (i.e., trait plasticity) of various shoot and root traits to SD_1/2cut_, RS_1/2cut_, and SR_1/2cut_, we calculated all the parameters using Log response ratios (LRR). The LRR was calculated as follows:

(1) LRR=Ln(T/treatmentT)control

where T_control_ represented the trait values in the control and T_treatment_ represented the trait values in the other three treatments.

To detect the additive effect of SD_1/2cut_ and RS_1/2cut_, moreover, we calculated the predicted LRRs (i.e., LRR_predicted_) of SR_1/2cut_ by summing the corresponding LRRs in the treatments of SD_1/2cut_ and RS_1/2cut_. They were additive effects when there were significant differences (*P* < 0.05) between the observed (LRRs in treatment of SR_1/2cut_, LRR_observed_) and predicted (LRR_predicted_) values. Meanwhile, the relative change ratios (RCs) of LRRs between LRR_observed_ and LRR_predicted_ were calculated as follows:

(2) RC=LRR/observedLRRpredicted

The allometric scaling among some key phenotypic traits of *L. chinensis* shoots and roots was assessed using the package “Standardized Major Axis Tests and Routines [(S)MATR].” Model Type II regression was used to determine the slope (a = scaling exponent) and y-intercept (log_10_b, where b is the allometric constant) of the log-log linear relationship. When the regressions under comparison had common slopes but different y-intercepts, the difference in the y-intercepts was inferred to underlie significant differences between the common slopes obtained under different treatments of CK, SD_1/2cut_, RS_1/2cut_, and SR_1/2cut_.

Kolmogorov–Smirnov tests were used to assess the assumption of normality of the data such as remaining and accumulated biomass, leaf and root phenotypic traits, and photosynthetic characteristics. Using IBM SPSS Statistics 25 (IBM Corporation, Armonk, NY, United States), differences in these indicators under different treatments were evaluated by a one-way analysis of variance (ANOVA). Meanwhile, the interactive effects of treatments and measurement time were evaluated by two-way ANOVA. Pearson’s correlation coefficient was used to evaluate the possible relationship between different indicators.

## Results

### Growth Rate and Shoot/Root Biomass Production

**FIGURE 1:**
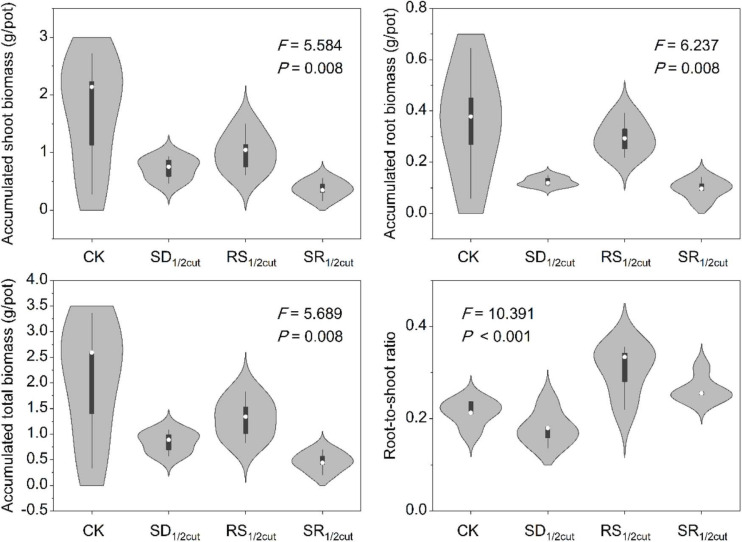
Effects of shoot defoliation and root severing on biomass accumulation and allocation of *Leymus chinensis*. CK, control; SD_1/2cut_, shoot defoliation; RS_1/2cut_, root severing; SR_1/2cut_, shoot defoliation and root severance.

**FIGURE 2:**
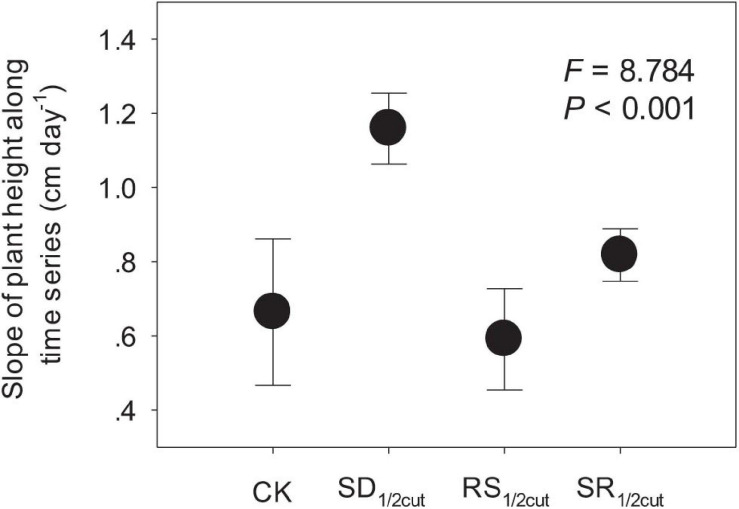
Effects of shoot defoliation and root severance on the growth rate (the slope of plant height along time series) of *Leymus chinensis*. CK, control; SD_1/2cut_, shoot defoliation; RS_1/2cut_, root severing; SR_1/2cut_, shoot defoliation and root severance.

Both remaining and accumulated biomass were negatively influenced by SD_1/2cut_, RS_1/2cut_, and SR_1/2cut_ (*P* < 0.05, Figure 1, Supplementary Figure 2, and Supplementary Table 1). The biomass accumulation was lower in RS_1/2cut_ than SD_1/2cut_ (*P* < 0.05; Figure 1). Compared to the roots, biomass accumulations of shoots had lower and higher reductions under SD_1/2cut_ and RS_1/2cut_, respectively, leading to a slight decrease (*P* > 0.05) and a significant increase (*P* < 0.05) in the root-to-shoot ratios respectively, (Figure 1). The remaining biomass was largely dependent on the proportion of defoliated or severed biomass in shoots (*P* < 0.05), not roots (*P* > 0.05, Supplementary Figure 3). Individual growth rate significantly varied among the treatments (*P* < 0.05), especially resulting in a significant increase in SD_1/2cut_ (*P* < 0.05, Figure 2 and Supplementary Figure 4). The growth rates, represented by slopes of plant height along a time series, were correlated with final (*P* < 0.05) rather than initial (*P* = 0.19) plant height (Supplementary Figure 5). In addition, a significant decrease in the major root and shoot phenotypic traits was observed for SD_1/2cut_, RS_1/2cut_, and SR_1/2cut_ treatments (*P* < 0.05), except for specific leaf area and specific root length (Supplementary Figures 6, 7 and Supplementary Table 2). Notably, specific leaf area increased with increasing root severance rather than shoot defoliation (*P* < 0.05, Supplementary Figure 6), whereas specific root length increased with increasing shoot defoliation but not by root severance (*P* < 0.05, Supplementary Figure 7).

### LRR and Trait Variations

**FIGURE 3:**
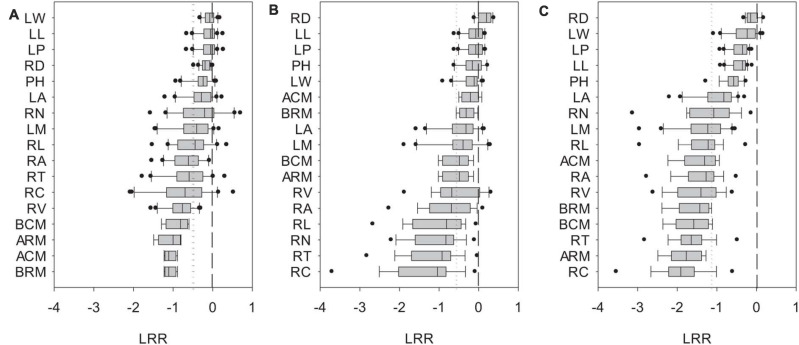
Ln-based response ratios (LRR) of shoot and root phenotypic traits of *Leymus chinensis* in response to **(A)** shoot defoliation, **(B)** root severing, and **(C)** shoot defoliation and root severance. Short and long dotted lines represent the mean value of response ratios of various phenotypic traits and the zero line. PH, plant height; LM, single leaf weight; LA, leaf area; LL, leaf length; LW, leaf width; LP, leaf perimeter; RL, total root length; RA, total root surface area; RV, total root volume; RD, average root diameter; RN, root tip number; RT, root tip forks; RC, root tip crossings; ARM, remaining aboveground biomass; BRM, remaining belowground biomass; BCM, aboveground biomass accumulation; ACM, belowground biomass accumulation.

**FIGURE 4:**
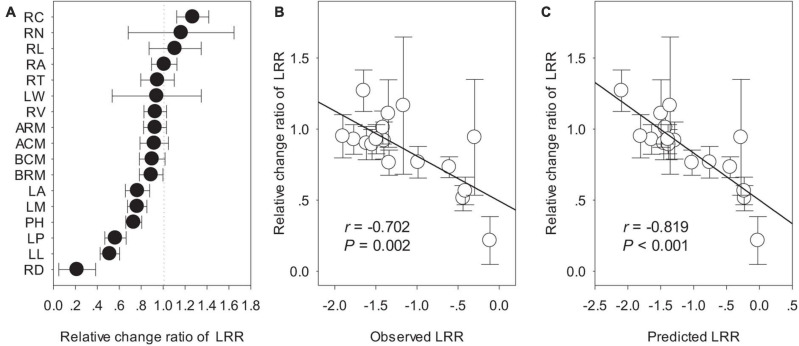
The relative change of ln response ratios (LRRs) between observed and predicted values: **(A)** ordering of relative change of LRRs; **(B)** linear relationship of observed values with the relative change of LRRs; **(C)** linear relationship of predicted values with the relative change of LRR. The observed values were the LRRs in the treatment of shoot defoliation and root severing (SR_1/2cut_), whereas predicted values were calculated by the LRRs in separate treatments of shoot defoliation (SD_1/2cut_) and root severing (RS_1/2cut_). PH, plant height; LM, single leaf weight; LA, leaf area; LL, leaf length; LW, leaf width; LP, leaf perimeter; RL, total root length; RA, total root surface area; RV, total root volume; RD, average root diameter; RN, root tip number; RT, root tip forks; RC, root tip crossings; ARM, remaining aboveground biomass; BRM, remaining belowground biomass; BCM, aboveground biomass accumulation; ACM, belowground biomass accumulation.

**FIGURE 5:**
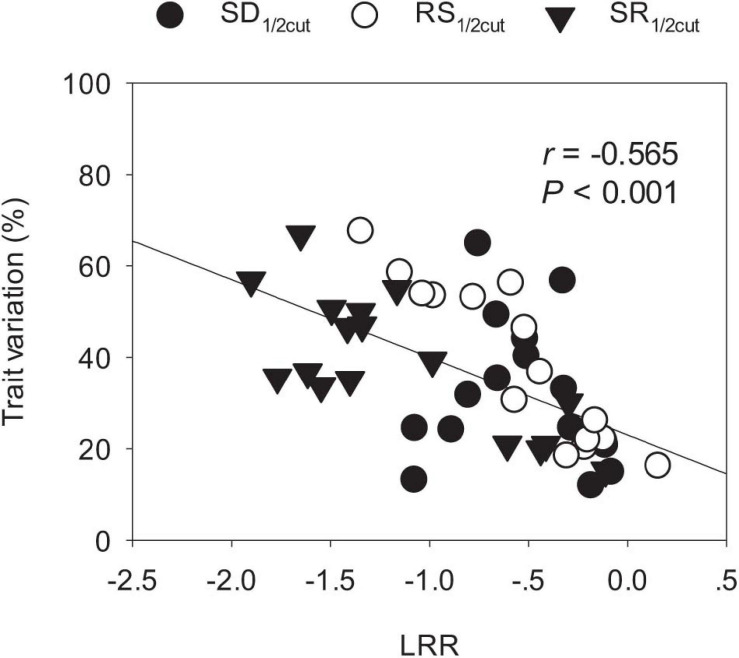
Linkages of ln response ratios (LRR) and trait variations among shoot and root phenotypic traits of *Leymus chinensis*. SD_1/2cut_, shoot defoliation; RS_1/2cut_, root severing; SR_1/2cut_, shoot defoliation and root severance.

Biomass and phenotypic traits of *L. chinensis* were negatively influenced by SD_1/2cut_ and RS_1/2cut_ (*P* < 0.05, Supplementary Tables 1, 2) treatments, with differences in sensitivity ranging more than 10-fold (Figure 3). Biomass-related indicators and root traits had higher LRRs than shoot phenotypic in the SD_1/2cut_ treatment compared to the control (Figure 3A). In contrast, negative LRRs were obtained for root traits in the RS_1/2cut_ treatment, followed by biomass-related indicators and shoot phenotypic traits (Figure 3B). Moreover, the interactive effects of SD_1/2cut_ and RS_1/2cut_ were relatively weak (*P* > 0.05), except for specific root length (*P* = 0.003, Supplementary Tables 1, 2). Notably, SR_1/2cut_ resulted in higher LRRs of all indicators than in both the SD_1/2cut_ and RS_1/2cut_ treatments (Figure 3C). In response to SR_1/2cut_, the sensitivities among *L. chinensis* indicators were as follows: root phenotypic traits > biomass-related indicators > shoot phenotypic traits (Figure 3C). The observed LRRs of SR_1/2cut_ were significantly correlated with predicted LRRs (*P* < 0.05), which were calculated by determining the LRRs of SD_1/2cut_ and RS_1/2cut_ (Supplementary Figure 8). However, the relative change ratios between observed and predicted LRRs dramatically varied with plant trait and were less than 1.0 in the majority of measured indicators, except for the three root traits (Figure 4). Additionally, trait variations within each treatment were contrastingly different among various shoot and root traits (Supplementary Table 3) and were highly correlated with trait plasticity (*P* < 0.01, Figure 5).

### Allometric Scaling Among Plant Traits

There was a significant positive relationship between log shoot traits and log root traits of *L. chinensis* within all pots across 15 of the SMA regressions (*P* < 0.05, Supplementary Figure 9). The allometric slopes for plant height with leaf perimeter (slope = 0.89, *P* = 0.03) and leaf mass per area (slope = 0.52, *P* < 0.01) were numerically less than 1.0 (Table 1). Among these measures, 13 of the 15 bivariate relationships had slopes significantly higher than 1.0 (*P* < 0.05, Table 1). Root traits generally had higher allometric slopes when plotted with plant height (2.06–2.23) than shoot phenotypic traits (Table 1). Similarly, the slopes of the SMA regressions between shoot traits (*x*-axis) and root traits (*y*-axis) were more than 1.0 (*P* < 0.05, Table 1). In addition, the shoot defoliation and root severing treatments resulted in heterogeneity of allometric slopes in the bivariate relationship between plant height and leaf area (*P* < 0.05, Supplementary Table 4). The SMA tests of the other 14 bivariate relationships showed that there were common slopes (*P* > 0.05) across the four treatments (Supplementary Table 4). However, there were significant shifts along the common slope of this bivariate relationships (*P* < 0.05, Supplementary Table 4).

**TABLE 1:** Standardized major axis regression slopes and intercepts with 95% confidence intervals (shown in brackets) for log-log transformed relationships among some key phenotypic traits of *Leymus chinensis* shoots and roots.

**Log-Y**	**Log-X**	***R*^2^**	***P*-value**	**Allometric slope**		**Allometric intercept**	
				**Mean value**	***P*-value**	**Mean value**	***P*-value**
LA	PH	0.80	<0.001	1.72 [1.55, 1.91]	<0.001	–1.64 [–1.89, –1.39]	<0.001
LP	PH	0.78	<0.001	0.89 [0.79, 0.99]	0.028	0.33 [0.20, 0.46]	<0.001
LMA	PH	0.60	<0.001	0.52 [0.45, 0.60]	<0.001	–3.12 [–3.23, –3.02]	<0.001
RL	PH	0.54	<0.001	2.14 [1.83, 2.50]	<0.001	–0.57 [–1.03, –0.10]	0.018
RA	PH	0.63	<0.001	2.06 [1.79, 2.37]	<0.001	–1.54 [–1.94, –1.14]	<0.001
RN	PH	0.44	<0.001	2.23 [1.88, 2.64]	<0.001	–0.12 [–0.65, 0.42]	0.663
RL	LA	0.72	<0.001	1.24 [1.10, 1.40]	0.001	1.47 [1.35, 1.59]	<0.001
RA	LA	0.76	<0.001	1.20 [1.07, 1.34]	0.002	0.43 [0.32, 0.53]	<0.001
RN	LA	0.65	<0.001	1.29 [1.13, 1.48]	<0.001	2.00 [1.86, 2.15]	<0.001
RL	LP	0.57	<0.001	2.42 [2.08, 2.81]	<0.001	–1.37 [–1.94, –0.79]	<0.001
RA	LP	0.60	<0.001	2.33 [2.02, 2.69]	<0.001	–2.31 [–2.84, –1.78]	<0.001
RN	LP	0.51	<0.001	2.52 [2.14, 2.95]	0.004	–0.95 [–1.58, –0.31]	0.004
RL	LMA	0.32	<0.001	4.10 [3.40, 4.95]	<0.001	12.23 [10.36, 14.09]	<0.001
RA	LMA	0.40	<0.001	3.95 [3.31, 4.72]	<0.001	10.80 [9.10, 12.49]	<0.001
RN	LMA	0.27	<0.001	4.27 [3.51, 5.19]	<0.001	13.20 [11.18, 15.22]	<0.001

*PH, plant height; LA, leaf area; LP, leaf perimeter; LMA, leaf mass per area; RL, total root length; RA, total root surface area; RN, root tip number.*

### Photosynthetic Responses to Treatments

**FIGURE 6:**
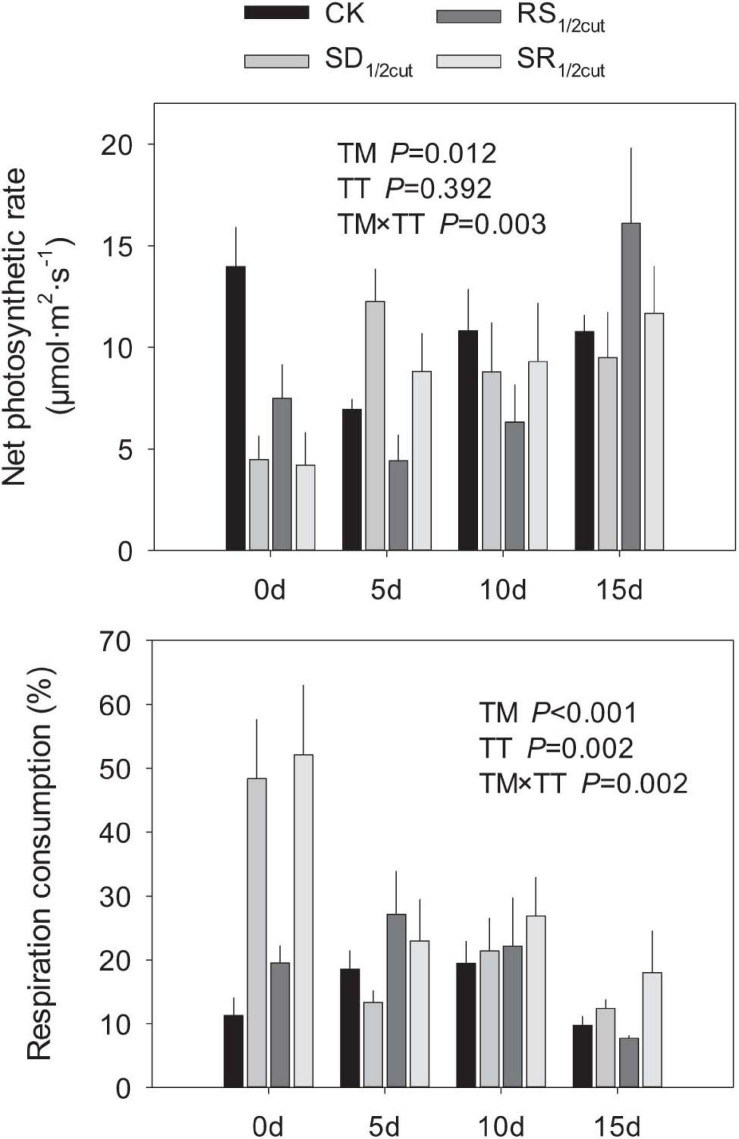
Effects of shoot defoliation and root severing on net photosynthetic rate and respiration consumption of *Leymus chinensis* after the second cycle of treatment. CK, control; SD_1/2cut_, shoot defoliation; RS_1/2cut_, root severing; SR_1/2cut_, shoot defoliation and root severing; TM, treatment; TT, time after treating.

Leaf photosynthetic rates were significantly influenced by the interactions between the experimental treatment and time after treatment application (*P* < 0.05, Figure 6 and Supplementary Figure 10). The SD_1/2cut_, RS_1/2cut_, and SR_1/2cut_ treatments significantly decreased both net and gross photosynthetic rates on the first day of the treatment (*P* < 0.05, Figure 6 and Supplementary Figure 10). In contrast, *L. chinensis* had higher respiration rates when shoots were defoliated rather than when roots were severed (*P* < 0.05, Supplementary Figure 10). However, the photosynthetic and respiration rates gradually restored over time, with no difference between the four treatments on the 15th day after treatments were applied (*P* < 0.05, Figure 6 and Supplementary Figure 10). In consequence, the respiration consumption proportion was rapidly increased (*P* < 0.05) in the first day of the treatment and the consumption proportion gradually decreased with time from the date of treatment (Figure 6). In addition, the photosynthetic and respiration rates of leaves were highly correlated with plant height (*P* < 0.05, Supplementary Figure 11).

## Discussion

Using a trait-based approach, we experimentally tested defoliation-induced legacy effects on the growth and persistence of the perennial grass species *L. chinensis*. Our results show evidence for the shoot and root growth feedback loop (hereafter also shoot-root feedback loop) influenced by defoliation, which may occur via mowing and grazing (Liu et al., 2015; Yang et al., 2020). In addition, our results suggested that the shoot–root feedback loop induced by defoliation was mediated by disproportionate response and allometry of plant functional traits. We found that the effect of experimental defoliation and root severance on trait plasticity of *L. chinensis* was sub-additive, may resulting in a buffering effect on plant performances under grazing or mowing. Overall, our study established that feedback between different plant organs contributes toward defoliation legacy effects, and has implications on the growth and persistence of the perennial grass species under intensifying utilization.

### Reciprocal Feedback Between Shoots and Roots

For perennial plant species, the interaction between above and belowground organs are critical to both individual growth in the current growing season and population persistence in the next year (Briske and Richards, 1995; Zong and Shi, 2019); thus, this may be the determining factor for the temporal stability of the whole ecosystem (Zhang et al., 2017; Xu et al., 2020). Two-rounds of shoot defoliation directly reduced the remaining and accumulated biomass by 64.66 and 57.51%, and indirectly reduced the root biomass by 60.11% (Figure 1 and Supplementary Figure 2). The reduction of root biomass may be due to the defoliation-induced reallocation of carbohydrate resources (Baptist et al., 2013; Wang et al., 2019). Our results suggested that root severance directly reduced 24.92 and 17.58% of the remaining and accumulated root biomass, and indirectly reduced 40.49% of shoot biomass (Figure 1 and Supplementary Figure 2). These finding clearly indicated that root severance generated a greater decrease in shoot biomass than root biomass. Together, these findings indicated that defoliation-induced reciprocal feedback between shoots and roots would generate legacy effects on future plant growth. Indeed, previous studies have shown that external disturbance can produce legacy effects via changes in soil abiotic properties and microorganisms in plant tissue and soil (Cuddington, 2011; Chen T. et al., 2018; Barthelemy et al., 2019). However, our finding of reciprocal feedback from different organs furthers our understanding of legacy effects independent from those generated by the fluctuations in the external environment.

In this study, both shoot defoliation and root severance generated under-compensatory growth, that is, the regenerated biomass was less than the lost biomass. The under-compensatory growth, rather than equal− or over-compensatory growth, might be caused by the high intensity of simulated defoliation and root severance. A previous study explicitly verified that over-and under-compensatory growth occurred in moderate and heavy treatments of grazing or mowing, respectively (Ma et al., 2020). In our experimental manipulation, half of the existing biomass was removed by twice, inducing strong adverse effects on plant growth capacity. Therefore, our results suggest that heavy, continuous grazing is detrimental to the plant growth not only via the direct effects of under-compensatory growth of shoot but also the indirect effects of root plasticity, finally impacting the sustainable utilization of grasslands.

### Shoot–Root Interaction Determines Biomass Production

Although shoots are directly affected by defoliation (Liu et al., 2018), we found that the biomass of the root had a stronger response to defoliation. The decline in root biomass might be triggered by the tight coupling mechanism between different plant organs, which has been reported in previous studies (Wang et al., 2010; Ma and Wang, 2021). It is intriguing that roots rather than shoots had a greater decline in biomass when *L. chinensis* was subjected to shoot defoliation, leading to a much lower root-to-shoot ratio in defoliated plants than that in undisturbed plants. One possible explanation for this observation is that plants may enhance the translocation of assimilates to aboveground rather than belowground organs (Gao et al., 2008; Esmaeili et al., 2009; Wang et al., 2019). Our findings support this explanation since we found that defoliation significantly enhanced the growth rate (the slopes of plant growth along time series) of *L. chinensis* by approximately 75% (from 0.66 cm day^–1^ in undisturbed plants to 1.16 cm day^–1^ in defoliated plants. Following defoliation, this shoot tissue regeneration can be dramatically enhanced to compensate for biomass loss, whereas the root growth was greatly suppressed. Further, our photosynthetic measurements also supported this speculation. Immediately following defoliation, the gross photosynthetic rates were markedly suppressed, whereas the respiration rates were greatly improved. However, these trends were reversed during the restoration process, indicating that photosynthetic rates were greatly improved, while respiration was reduced. This could be ascribed to the regulation of the partitioning of new photosynthate carbon in response to plant tissue damage and biomass loss (Schierenbeck et al., 1994; Wang et al., 2019).

We found that root severance triggered a greater decrease in shoots than that in roots, resulting in a significant increase in the root-to-shoot ratio, which may be explained by the reallocation of photosynthate carbon to offset the lost root biomass. This finding suggests that root plasticity induced by shoot defoliation can generate a severe cascading reaction of plant growth in the following stage. Given that heavy grazing or frequent mowing tend to make the roots shallower (Kitchen et al., 2009), this finding that roots had more sensitive response than shoots to root severance (i.e., asymmetric response) can provide new insights for understanding the mechanism of grassland degradation. In general, root biomass highly relies on the changes in root traits (Roumet et al., 2008). Across the root traits, root volume, root surface area, and root diameter were tightly correlated with the changes in root biomass after severing treatment. Thus, an increased plasticity of root traits could lead to a decrease in the stability of root biomass.

### Disproportionate Response and Allometry of Plant Traits

The linkage of functional traits with plant adaptation and ecological functioning have been addressed in previous studies (Garnier and Navas, 2012; van der Merwe et al., 2021). Results from the present study provided a new addition to this trait-based approach from the perspective of trait plasticity under defoliation. Although all traits negatively responded to shoot defoliation and root severance, the extent of trait plasticity dramatically (by approximately 10-fold) among the stable and sensitive traits. In general, across all plant traits, root traits were more sensitive than shoot traits in both the shoot defoliation and root severing treatments. Based on the SMA approach, we further showed that shoot/root asymmetry in plants with both shoot defoliation and root severing was allometric according to the regressions of log10-transformed traits. In agreement with studies on grassland species (Luo et al., 2013; Ma and Wang, 2021), the allometric slopes of shoots and roots were significantly different, indicating that *L. chinensis* traits did not meet the isometric prediction.

In general, the allometric slopes in bivariate relationships of aboveground traits (*x*-axis) versus belowground traits (*y*-axis), which varied from 1.20 to 4.27, were markedly higher than 1.0. In terms of the trait-specific allometry between above and belowground organs, shoot traits had relatively higher allometric slopes with root tip number and total root length. We surmise that this result is most likely related to the species growth phase and experimental methodology (Gedroc et al., 1996; Wang et al., 2010). First, plant age can potentially determine the allometric scaling between above and belowground organs. For example, Husáková et al. (2018) found that plant species generally allocated disproportionally more carbon photosynthate to aboveground organs as seedlings than as adults. In this study, the allometric scaling was assessed in 65-day-old plants, which corresponds to the rapid growth phase of *L. chinensis*; this may stimulate a higher biomass allocation in shoots than roots. Second, plants preferentially promoted the growth of aboveground than belowground organs because the water and nutrient resources were supplied in sufficient amounts in hydroponics with Hoagland’s nutrient solution (Ren et al., 2017).

### Sub-Additive Effect of Shoot Defoliation and Root Severance

We demonstrated that the LRR of all shoot or root traits in RS_1/2cut_ were greater than either SD_1/2cut_ or RS_1/2cut_. However, the findings also showed that there was no significant interactive effect on plant performance for either defoliation or root biomass removal. Of particular interest is that the combined effects of shoot and root biomass removal were sub-additive, that is, the LRRs of plant traits in SR_1/2cut_ was less than the sum of the independent effects of the two manipulations. This sub-additive effect can buffer the predicted negative feedback loop of non-adaptive plasticity in shoots and roots induced by defoliation (Arredondo and Johnson, 1999; Zhang et al., 2018), thereby preventing the worst−case outcomes of plant fitness. The observed sub-additive effects could arise from the enhancement of metabolic activity, such as photosynthesis, thus leading to a positive compensatory effect to cope with biomass loss (Richards and Caldwell, 1985; Iqbal et al., 2012). This is supported by our finding that photosynthetic and respiratory rates showed higher respiratory consumption and photosynthetic rates in SR_1/2cut_, which potentially stimulated key biological processes including biosynthesis, metabolism, and carbon remobilization.

In terms of trait-specific LRRs, our results showed that the relative change between observed and predicted LRRs, ranging from 0.22 to 1.27, meaning they varied greatly and were tight correlated with observed or predicted LRRs. This directly suggested that the sub-additive effects were dependent on plant traits. To our knowledge, our study is the first to report this. Comparatively, root traits had greater relative change between observed and predicted LRRs than shoot traits. We suspect the differences between shoots and roots can be partly explained by the trade-off and allometry of functional traits in response to disturbances (Li et al., 2016). For example, a recent study on *Artemisia* showed that an allometric strategy among leaves, stems, and roots was prevalent and was essential to optimize plant performance under environmental gradients (Liu et al., 2021). In the present study, considering that roots had higher plasticity than shoots, the stronger sub-additive effects of root traits may potentially buffer the negative effects of defoliation.

## Conclusion

Overall, this study presents strong evidence that defoliation can produce a robust feedback loop for shoot and root growth, potentially generating observable legacy effects on plant performance at a later growth stage or even in following growing seasons. This shoot–root feedback is mediated by allometry among plant traits. Our findings showed that the combined effects of shoot defoliation and root severing were less than the sum of their independent effects but statistically greater than the effect of each of the two manipulations. In consequence, this sub-additive effect can buffer the negative influences of shoot–root feedback on plant performance induced by defoliation. Considering the existing knowledge of legacy effects due to land use, our findings provide new insight for this topic from the reciprocal feedback between shoots and roots of grassland species affected by a high-intensity of defoliation.

## Data Availability Statement

The original contributions presented in the study are included in the article/Supplementary Material, further inquiries can be directed to the corresponding author/s.

## Author Contributions

XL and JS designed the research and wrote first draft of the manuscript. XL, ZZ, FG, and JD performed the research. XL, ZZ, and FG analyzed the data. All authors contributed critically to revisions and gave final approval for publication.

## Conflict of Interest

The authors declare that the research was conducted in the absence of any commercial or financial relationships that could be construed as a potential conflict of interest.

## Publisher’s Note

All claims expressed in this article are solely those of the authors and do not necessarily represent those of their affiliated organizations, or those of the publisher, the editors and the reviewers. Any product that may be evaluated in this article, or claim that may be made by its manufacturer, is not guaranteed or endorsed by the publisher.

## Supplementary Material

Supplementary Figure 1 Scheme of the experimental setup. Root or shoot removal was conducted in plants grown hydroponically. SD_1/2cut_, shoot defoliation; RS_1/2cut_, root severing; SR_1/2cut_, shoot defoliation and root severing.

Supplementary Figure 2 Biomass composition of the remaining organ and the removed organ induced by shoot defoliation **(A1–D1)** or root severing **(A2–D2)** of *Leymus chinensis*. **(A1,B1)** Control; **(A2,B2)** shoot defoliation; **(A3,B3)** root severing; and **(A4,B4)** shoot defoliation and root severing. T1 and T2 mean the first and second cutting.

Supplementary Figure 3 Relationships of the proportion of defoliated or severed biomass with the remaining biomass in the shoot **(A1,A2)**, root **(B1,B2)** and overall **(C1,C2)** with **(A1–C1)** or without **(A2–C2)** a control treatment.

Supplementary Figure 4 Plant height of *Leymus chinensis* across the four treatments along a time series. CK, control; SD_1/2cut_, shoot defoliation; RS_1/2cut_, root severing; SR_1/2cut_, shoot defoliation and root severing.

Supplementary Figure 5 Relationship of the slope of plant height with the initial and final plant height of *L. chinensis* along a time series.

Supplementary Figure 6 Effects of defoliation and root severing on *L. chinensis* leaf traits. CK, control; SD_1/2cut_, shoot defoliation; RS_1/2cut_, root severing; SR_1/2cut_, shoot defoliation and root severing.

Supplementary Figure 7 Effects of defoliation and root severing on *L. chinensis* root traits. CK, control; SD_1/2cut_, shoot defoliation; RS_1/2cut_, root severing; SR_1/2cut_, shoot defoliation plus root severing.

Supplementary Figure 8 The predicted ln-based response ratios (LRRs) **(A)** and their relationship with observed LRRs **(B)**. The observed values were the LRRs in the shoot defoliation and root severing treatment (SR_1/2cut_), whereas the predicted values were calculated by the LRRs in separate treatments of shoot defoliation (SD_1/2cut_) and root severing (RS_1/2cut_). PH, plant height; LM, single leaf weight; LA, leaf area; LL, leaf length; LW, leaf width; LP, leaf perimeter; RL, total root length; RA, total root surface area; RV, total root volume; RD, average root diameter; RN, root tip number; RT, root tip forks; RC, root tip crossings; ARM, remaining aboveground biomass; BRM, remaining belowground biomass; BCM, aboveground biomass accumulation; ACM, belowground biomass accumulation.

Supplementary Figure 9 Relationships of log-log transformed shoot and root traits of *L. chinensis*. PH, plant height; LA, leaf area; LP, leaf perimeter; LMA, leaf mass per area; RL, total root length; RA, total root surface area; RN, root tip number.

Supplementary Figure 10 Effects of shoot defoliation and root severing on the gross photosynthetic rate and respiration rate of *L. chinensis* after the second cycle of treatment across the time series. CK, control; SD_1/2cut_, shoot defoliation; RS_1/2cut_, root severing; SR_1/2cut_, shoot defoliation and root severing; TM, treatment; TT, time after treating.

Supplementary Figure 11 Linking plant size with the photosynthetic and respiration rates.

Supplementary Table 1 Effects of shoot defoliation (SD_1/2cut_), root severing (RS_1/2cut_), and their interactions (SD_1/2cut_ × RS_1/2cut_) on the biomass of *L. chinensis*. ARM, remaining aboveground biomass; BRM, remaining belowground biomass; TRM, remaining total biomass; RRS, root-to-shoot ratio of remaining biomass; BCM, aboveground biomass accumulation; ACM, belowground biomass accumulation; TCM, total biomass accumulation; CRS, root-to-shoot ratio of accumulated biomass.

Supplementary Table 2 Effects of shoot defoliation (SD_1/2cut_), root severing (RS_1/2cut_), and their interactions (SD_1/2cut_ × RS_1/2cut_) on phenotypic traits of *L. chinensis* shoots and roots. PH, plant height; LA, leaf area; LM, single leaf weight; SLA, specific leaf area; LL, leaf length; LW, leaf width; LP, leaf perimeter; RL, total root length; SRL, specific root length; RA, total root surface area; RD, average root diameter; RV, total root volume; RN, root tip number; RT, root tip forks; RC, root tip crossings.

Supplementary Table 3 *L. chinensis* shoot and root trait variations (%) in the control (CK), shoot defoliation (SD_1/2cut_), root severing (RS_1/2cut_), and shoot defoliation and root severing (SR_1/2cut_) treatments. PH, plant height; LM, single leaf weight; LA, leaf area; LL, leaf length; LW, leaf width; LP, leaf perimeter; RL, total root length; RA, total root surface area; RV, total root volume; RD, average root diameter; RN, root tip number; RT, root tip forks; RC, root tip crossings; ARM, remaining aboveground biomass; BRM, remaining belowground biomass; BCM, aboveground biomass accumulation; ACM, belowground biomass accumulation.

Supplementary Table 4 Standardized major axis regression (SMA) slopes for log-log transformed relationships among some key phenotypic traits of *L. chinensis* shoots and roots in the four treatments. In several of the bivariate cases, SMA tests for common slopes reveal no significant differences among the four treatment groups (i.e., *P* > 0.05). In such cases, common slopes for the bivariate relationships were shown. Where there was a common slope, significant shifts (*P* < 0.05; labeled “yes” in the table) along a common slope are also indicated. CK, control; SD_1/2cut_, shoot defoliation; RS_1/2cut_, root severing; SR_1/2cut_, shoot defoliation and root severing. PH, plant height; LA, leaf area; LP, leaf perimeter; LMA, leaf mass per area; RL, total root length; RA, total root surface area; RN, root tip number.
