# Supplementary material for: Shoot–Root Interplay Mediates Defoliation-Induced Plant Legacy Effect
Source: Front Plant Sci. 2021 Aug 5;12:684503. doi: 10.3389/fpls.2021.684503 (PMC8374956; doi:10.3389/fpls.2021.684503)
Supplement: Supplementary Figure 1 — Scheme of the experimental setup. Root or shoot removal was conducted in plants grown hydroponically. SD1/2cut, shoot defoliation; RS1/2cut, root severing; SR1/2cut, shoot defoliation and root severing. [file Data_Sheet_1.docx]

**Supplementary Material**


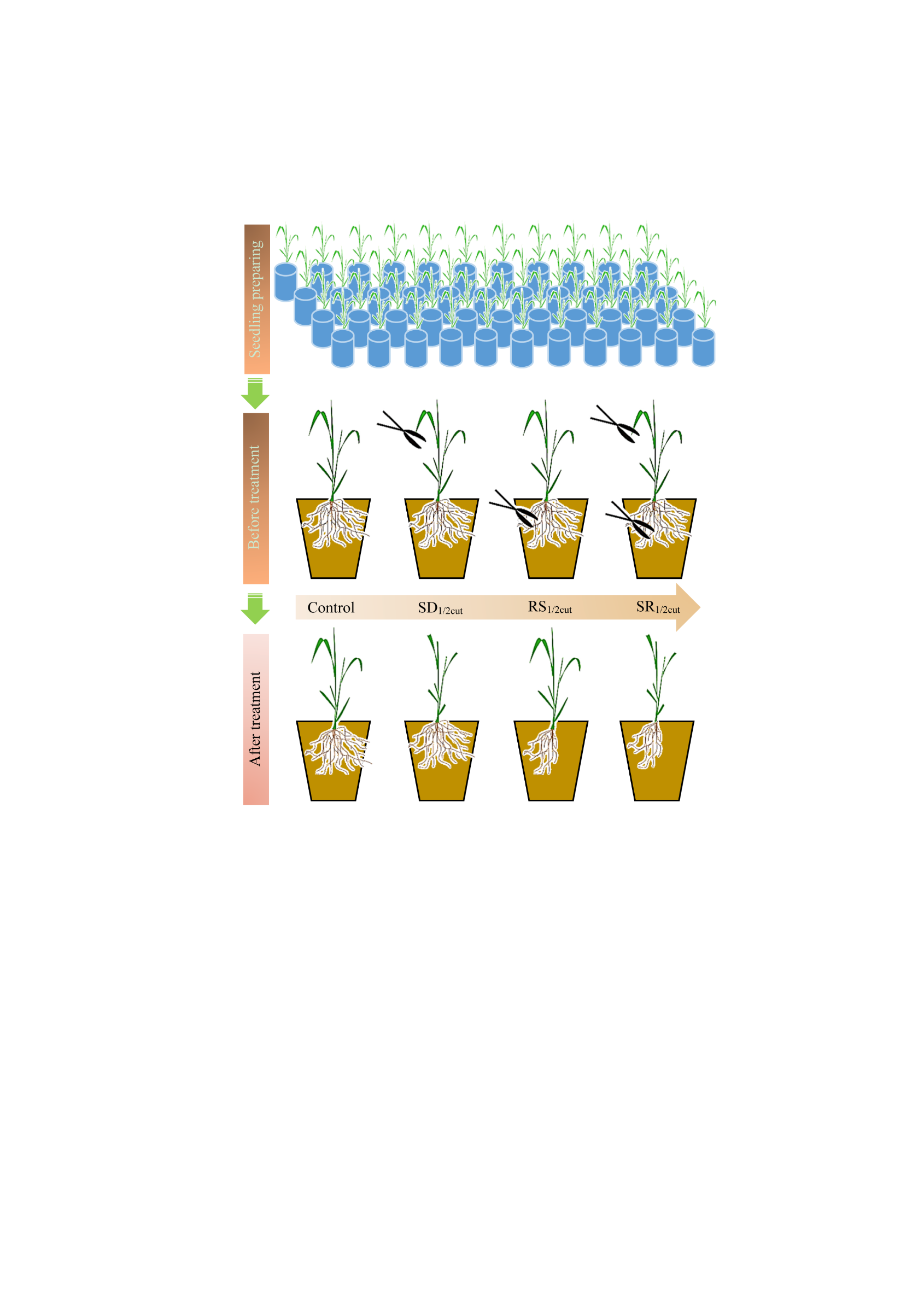


**Supplementary Figure 1**. Scheme of the experimental setup. Root or shoot removal was conducted in plants grown hydroponically. SD_1/2cut_, shoot defoliation; RS_1/2cut_, root severing; SR_1/2cut_, shoot defoliation and root severing.

**Supplementary Figure 2**. Biomass composition of the remaining organ and the removed organ induced by shoot defoliation (a1, b1, c1, d1) or root severing (a2, b2, c2, d2) of *Leymus chinensis*. a1 and b1, control; a2 and b2, shoot defoliation; a3 and b3, root severing; a4 and b4, shoot defoliation and root severing. T1 and T2 mean the first and second cutting.

**Supplementary Figure 3**. Relationships of the proportion of defoliated or severed biomass with the remaining biomass in the shoot (a1, a2), root (b1, b2) and overall (c1, c2) with (a1, b1, c1) or without (a2, b2, c2) a control treatment.

**Supplementary Figure 4**. Plant height of *Leymus chinensis* across the four treatments along a time series. CK, control; SD_1/2cut_, shoot defoliation; RS_1/2cut_, root severing; SR_1/2cut_, shoot defoliation and root severing.

**Supplementary Figure 5**. Relationship of the slope of plant height with the initial and final plant height of *L. chinensis* along a time series.

**Supplementary Figure 6**. Effects of defoliation and root severing on *L. chinensis* leaf traits. CK, control; SD_1/2cut_, shoot defoliation; RS_1/2cut_, root severing; SR_1/2cut_, shoot defoliation and root severing.

**Supplementary Figure 7**. Effects of defoliation and root severing on *L. chinensis* root traits. CK, control; SD_1/2cut_, shoot defoliation; RS_1/2cut_, root severing; SR_1/2cut_, shoot defoliation plus root severing.

**Supplementary Figure 8**. The predicted ln-based response ratios (LRRs) (a) and their relationship with observed LRRs (b). The observed values were the LRRs in the shoot defoliation and root severing treatment (SR_1/2cut_), whereas the predicted values were calculated by the LRRs in separate treatments of shoot defoliation (SD_1/2cut_) and root severing (RS_1/2cut_). PH, plant height; LM, single leaf weight; LA, leaf area; LL, leaf length; LW, leaf width; LP, leaf perimeter; RL, total root length; RA, total root surface area; RV, total root volume; RD, average root diameter; RN, root tip number; RT, root tip forks; RC, root tip crossings; ARM, remaining aboveground biomass; BRM, remaining belowground biomass; BCM, aboveground biomass accumulation; ACM, belowground biomass accumulation.

**Supplementary Figure 9**. Relationships of log-log transformed shoot and root traits of *L. chinensis*. PH, plant height; LA, leaf area; LP, leaf perimeter; LMA, leaf mass per area; RL, total root length; RA, total root surface area; RN, root tip number.

**Supplementary Figure 10**. Effects of shoot defoliation and root severing on the gross photosynthetic rate and respiration rate of *L. chinensis* after the second cycle of treatment across the time series. CK, control; SD_1/2cut_, shoot defoliation; RS_1/2cut_, root severing; SR_1/2cut_, shoot defoliation and root severing; TM, treatment; TT, time after treating.

**Supplementary Figure 11**. Linking plant size with the photosynthetic and respiration rates.

**Supplementary Table 1**. Effects of shoot defoliation (SD_1/2cut_), root severing (RS_1/2cut_), and their interactions (SD_1/2cut_ × RS_1/2cut_) on the biomass of *L. chinensis*. ARM, remaining aboveground biomass; BRM, remaining belowground biomass; TRM, remaining total biomass; RRS, root-to-shoot ratio of remaining biomass; BCM, aboveground biomass accumulation; ACM, belowground biomass accumulation; TCM, total biomass accumulation; CRS, root-to-shoot ratio of accumulated biomass.

| Variables | SD_1/2cut_ | |  | RS_1/2cut_ | |  | SD_1/2cut_ × RS_1/2cut_ | |
| --- | --- | --- | --- | --- | --- | --- | --- | --- |
|  | *F* | *P*-value |  | *F* | *P*-value |  | *F* | *P*-value |
| ARM | 14.36 | 0.002 |  | 4.24 | 0.056 |  | 0.71 | 0.412 |
| BRM | 17.37 | 0.001 |  | 1.65 | 0.217 |  | 0.23 | 0.637 |
| TRM | 15.18 | 0.001 |  | 3.77 | 0.07 |  | 0.62 | 0.443 |
| RRS | 0.13 | 0.724 |  | 9.87 | 0.006 |  | 0.21 | 0.653 |
| BCM | 11.51 | 0.004 |  | 4.77 | 0.044 |  | 0.48 | 0.497 |
| ACM | 17.82 | 0.001 |  | 0.77 | 0.393 |  | 0.12 | 0.735 |
| TCM | 12.78 | 0.003 |  | 3.88 | 0.066 |  | 0.41 | 0.532 |
| CRS | 5.02 | 0.04 |  | 26.11 | 0 |  | 0.06 | 0.818 |

**Supplementary Table 2**. Effects of shoot defoliation (SD_1/2cut_), root severing (RS_1/2cut_), and their interactions (SD_1/2cut_ × RS_1/2cut_) on phenotypic traits of *L. chinensis* shoots and roots. PH, plant height; LA, leaf area; LM, single leaf weight; SLA, specific leaf area; LL, leaf length; LW, leaf width; LP, leaf perimeter; RL, total root length; SRL, specific root length; RA, total root surface area; RD, average root diameter; RV, total root volume; RN, root tip number; RT, root tip forks; RC, root tip crossings.

| Variables | SD_1/2cut_ | |  | RS_1/2cut_ | |  | SD_1/2cut_ × RS_1/2cut_ | |
| --- | --- | --- | --- | --- | --- | --- | --- | --- |
|  | *F* | *P*-value |  | *F* | *P*-value |  | *F* | *P*-value |
| PH | 23.65 | <0.001 |  | 9.32 | 0.003 |  | 0.82 | 0.367 |
| LA | 11.14 | 0.001 |  | 17.89 | <0.001 |  | 0.32 | 0.571 |
| LM | 19.57 | <0.001 |  | 17.03 | <0.001 |  | 0.18 | 0.675 |
| SLA | 16.15 | <0.001 |  | 3.17 | 0.079 |  | 4.81 | 0.032 |
| LL | 10.70 | 0.002 |  | 11.03 | 0.001 |  | 3.16 | 0.080 |
| LW | 1.41 | 0.239 |  | 6.93 | 0.010 |  | 0.03 | 0.866 |
| LP | 9.08 | 0.004 |  | 9.67 | 0.003 |  | 2.28 | 0.135 |
| RL | 7.53 | 0.008 |  | 24.74 | <0.001 |  | 1.01 | 0.317 |
| SRL | 100.78 | <0.001 |  | 65.28 | <0.001 |  | 9.26 | 0.003 |
| RA | 17.95 | <0.001 |  | 19.67 | <0.001 |  | 1.13 | 0.292 |
| RD | 42.11 | <0.001 |  | 13.48 | <0.001 |  | 3.19 | 0.078 |
| RV | 31.49 | <0.001 |  | 11.93 | 0.001 |  | 0.67 | 0.414 |
| RN | 0.93 | 0.338 |  | 23.93 | <0.001 |  | 0.26 | 0.613 |
| RT | 9.37 | 0.003 |  | 27.29 | <0.001 |  | 1.71 | 0.195 |
| RC | 7.55 | 0.008 |  | 23.66 | <0.001 |  | 1.48 | 0.228 |

**Supplementary Table 3**. *L. chinensis* shoot and root trait variations (%) in the control (CK), shoot defoliation (SD_1/2cut_), root severing (RS_1/2cut_), and shoot defoliation and root severing (SR_1/2cut_) treatments. PH, plant height; LM, single leaf weight; LA, leaf area; LL, leaf length; LW, leaf width; LP, leaf perimeter; RL, total root length; RA, total root surface area; RV, total root volume; RD, average root diameter; RN, root tip number; RT, root tip forks; RC, root tip crossings; ARM, remaining aboveground biomass; BRM, remaining belowground biomass; BCM, aboveground biomass accumulation; ACM, belowground biomass accumulation.

| Variables | CK | SD_1/2cut_ | RS_1/2cut_ | SR_1/2cut_ |
| --- | --- | --- | --- | --- |
| ARM | 51.68 | 24.33 | 30.51 | 35.59 |
| BRM | 53.99 | 13.08 | 18.37 | 33.72 |
| ACM | 35.08 | 13.08 | 20.23 | 35.08 |
| BCM | 36.72 | 24.06 | 30.51 | 36.72 |
| PH | 37.23 | 24.45 | 26.03 | 20.89 |
| LA | 53.29 | 33.00 | 36.67 | 39.28 |
| LM | 53.63 | 40.14 | 46.30 | 46.99 |
| LL | 32.33 | 20.69 | 21.60 | 19.88 |
| LW | 36.81 | 14.81 | 21.85 | 30.07 |
| LP | 32.55 | 21.08 | 22.05 | 20.85 |
| RL | 69.73 | 44.02 | 53.39 | 49.89 |
| RA | 64.06 | 35.28 | 53.05 | 46.63 |
| RD | 15.89 | 11.86 | 16.12 | 15.25 |
| RV | 59.39 | 31.60 | 56.12 | 50.60 |
| RN | 75.28 | 56.68 | 53.82 | 54.93 |
| RT | 73.60 | 49.18 | 58.44 | 56.81 |
| RC | 82.99 | 64.79 | 67.50 | 66.90 |

**Supplementary Table 4**. Standardized major axis regression (SMA) slopes for log-log transformed relationships among some key phenotypic traits of *L. chinensis* shoots and roots in the four treatments. In several of the bivariate cases, SMA tests for common slopes reveal no significant differences among the four treatment groups (i.e., *P* > 0.05). In such cases, common slopes for the bivariate relationships were shown. Where there was a common slope, significant shifts (*P* < 0.05; labeled ‘yes’ in the table) along a common slope are also indicated. CK, control; SD_1/2cut_, shoot defoliation; RS_1/2cut_, root severing; SR_1/2cut_, shoot defoliation and root severing. PH, plant height; LA, leaf area; LP, leaf perimeter; LMA, leaf mass per area; RL, total root length; RA, total root surface area; RN, root tip number.

| Bivariate relationship | |  | CK | |  | SD_1/2cut_ | |  | RS_1/2cut_ | |  | SR_1/2cut_ | |  | Heterogeneity of slopes | | |
| --- | --- | --- | --- | --- | --- | --- | --- | --- | --- | --- | --- | --- | --- | --- | --- | --- | --- |
| Log-Y | Log-X |  | Slope | *P*-value |  | Slope | *P*-value |  | Slope | *P*-value |  | Slope | *P*-value |  | Common slope | *P*-value | shift? |
| LA | PH |  | 1.85 | <0.001 |  | 1.37 | 0.001 |  | 1.72 | 0.002 |  | 2.08 | <0.001 |  | / | 0.027 | / |
| LP | PH |  | 0.97 | 0.741 |  | 0.83 | 0.068 |  | 0.89 | 0.402 |  | 0.98 | 0.928 |  | 0.91 | 0.616 | Yes |
| LMA | PH |  | 0.47 | <0.001 |  | 0.47 | 0.001 |  | 0.54 | 0.001 |  | 0.64 | 0.034 |  | 0.52 | 0.614 | Yes |
| RL | PH |  | 2.23 | <0.001 |  | 1.67 | 0.006 |  | 2.54 | <0.001 |  | 2.32 | <0.001 |  | 2.15 | 0.416 | Yes |
| RA | PH |  | 2.20 | <0.001 |  | 1.40 | 0.068 |  | 2.26 | 0.001 |  | 2.24 | <0.001 |  | 2.04 | 0.194 | Yes |
| RN | PH |  | 2.28 | <0.001 |  | 2.11 | <0.001 |  | 2.31 | 0.001 |  | 2.88 | <0.001 |  | 2.32 | 0.694 | Yes |
| RL | LA |  | 1.20 | 0.101 |  | 1.22 | 0.260 |  | 1.48 | 0.018 |  | 1.12 | 0.490 |  | 1.24 | 0.625 | Yes |
| RA | LA |  | 1.19 | 0.100 |  | 1.02 | 0.898 |  | 1.31 | 0.110 |  | 1.08 | 0.600 |  | 1.16 | 0.711 | Yes |
| RN | LA |  | 1.23 | 0.117 |  | 1.54 | 0.008 |  | 1.34 | 0.088 |  | 1.38 | 0.053 |  | 1.36 | 0.714 | Yes |
| RL | LP |  | 2.30 | <0.001 |  | 2.01 | 0.002 |  | 2.87 | <0.001 |  | 2.37 | <0.001 |  | 2.39 | 0.604 | Yes |
| RA | LP |  | 2.27 | <0.001 |  | 1.69 | 0.017 |  | 2.56 | <0.001 |  | 2.28 | <0.001 |  | 2.22 | 0.510 | Yes |
| RN | LP |  | 2.34 | <0.001 |  | 2.54 | <0.001 |  | 2.61 | <0.001 |  | 2.93 | <0.001 |  | 2.57 | 0.845 | Yes |
| RL | LMA |  | 4.76 | <0.001 |  | 3.56 | <0.001 |  | 4.69 | <0.001 |  | 3.63 | <0.001 |  | 4.01 | 0.564 | Yes |
| RA | LMA |  | 4.70 | <0.001 |  | 2.98 | <0.001 |  | 4.18 | <0.001 |  | 3.50 | <0.001 |  | 3.75 | 0.449 | Yes |
| RN | LMA |  | 4.86 | <0.001 |  | 4.50 | <0.001 |  | 4.27 | <0.001 |  | 4.50 | <0.001 |  | 4.52 | 0.983 | Yes |
